# Referral for glaucoma surgery and types of surgery in different European regions in 2025, the EURACCUR study

**DOI:** 10.1007/s00417-026-07155-x

**Published:** 2026-03-26

**Authors:** Jose Martinez-de-la-Casa, Ingeborg Stalmans, Luís Abegão-Pinto, Fotis Topouzis, Marta Pazos, Heny Beckers, Ingride Januleviciene, Francesco Oddone, Karl Mercieca, Ana Miguel, Gauti Jóhannesson, Miriam Kolko, Mia Zorić Geber, Barbara Cvenkel, Francis Carbonaro, Nikola Babic, Javier Garcia-Bardera, Mehmed Ikinci, Aurija Kalasauskiene, Gloria Roberti, Jan Henrik Simonsen, Tena Križ, Adrian Mifsud, Constance Liegl, Gábor Holló

**Affiliations:** 1 Ophthalmology Service, Hospital Clinico San Carlos. Complutense University, Madrid, Spain; 2 Institute of Health Research, Hospital Clinico San Carlos (IdISSC), Madrid, Spain; 3 Department of Ophthalmology, University Hospitals, Leuven, Belgium; 4 Research Group Ophthalmology, Department of Neurosciences, KU Leuven, Leuven, Flanders Belgium; 5 Department of Ophthalmology, Visual Sciences Study Center, ULS Santa Maria. Visual Sciences Study Center, Faculty of Medicine of Lisbon University, Lisbon, Portugal; 6 Department of Ophthalmology, Medical School, Aristotle University of Thessaloniki, AHEPA Hospital, Thessaloniki, Greece; 7 Institute of Ophthalmology. Hospital Clínic Barcelona, Barcelona University, Institute of Health Research August Pi i Sunyer (IDIBAPS), Barcelona, Spain; 8 University Eye Clinic Maastricht, Maastricht University Medical Center+, Maastricht, the Netherlands; 9 Eye clinic of Lithuanian University of Health Sciences, Kaunas, Lithuania; 10 IRCCS-Fondazione Bietti, Rome, Italy; 11 University Eye Clinic Bonn, Bonn, Germany; 12 Glaucoma Department, Ophthalmology, Private Hospital of la Baie, Avranches, France; 13 Central University Hospital of Caen, Caen, France; 14 Department of Clinical Sciences, Ophthalmology, Umeå University, Umeå, Sweden; 15 Department of Clinical Neuroscience, Karolinska Institutet, St Eriks Eye Hospital, Stockholm, Sweden; 16 Department of Ophthalmology, University of Iceland, Reykjavik, Iceland; 17 Department of Ophthalmology, Aalborg University Hospital, Aalborg, Denmark; 18 Department of Drug Design and Pharmacology, University of Copenhagen, Copenhagen, Denmark; 19 Department of Ophthalmology, Rigshospitalet, Copenhagen, Denmark; 20 Eye Hospital Your Eye Doctors, Rungsted Kyst, Denmark; 21 Department of Ophthalmology, Sestre Milosrdnice University Hospital Center, Zagreb, Croatia; 22 Department of Ophthalmology, University Medical Centre Ljubljana, Ljubljana, Slovenia; 23 Faculty of Medicine, University of Ljubljana, Ljubljana, Slovenia; 24 Ophthalmic Department, Mater Dei Hospital, Msida, Malta; 25 Department of Twin Research, King’s College, London, UK; 26 Faculty of Medicine, University of Novi Sad, Novi Sad, Serbia; 27 University Eye Clinic, Clinical Center of Vojvodina, Novi Sad, Serbia; 28 Tutkimusz Ltd, Solymár, Hungary; 29 Ophthalmology Center, Prima Medica Health Centers, Budapest, Hungary

**Keywords:** European glaucoma practice, MIBS, MIGS, Trabeculectomy, Referral for glaucoma surgery

## Abstract

**Purpose:**

To investigate the characteristics of referral for glaucoma surgery and compare surgical practices in various European regions in 2025.

**Methods:**

Data of 300 eyes of 300 consecutive patients undergoing glaucoma surgery were analysed using a standardized questionnaire and compared between geographical regions. Glaucoma specialists from one centre per country provided data on demographics, glaucoma types, intraocular pressure (IOP), visual field and structural metrics, types of surgery and referral timeliness (timely, later than optimal, late).

**Results:**

The median (quartiles) age was 72 (64–78) years. Primary open angle glaucoma (52.0%) and pseudoexfoliative glaucoma (23.0%) were the most common glaucoma types. Median disease duration was 75 (28–144) months, preoperative IOP 22 (18–28) mmHg, mean deviation − 14.47 (− 21.60 to − 7.85) dB, and retinal nerve fibre layer thickness 60 (50–75) µm. Trabeculectomy (38.3%), minimally invasive bleb forming surgeries (28.3%), and drainage devices (11.3%) were the predominant procedures; 18.7% of the surgeries were combined with phacoemulsification. More severe disease was associated with filtering or drainage device surgery (*p* < 0.001). Referral was timely in 34.7%, later than optimal in 35.7%, and late in 29.7%. Late referrals were associated with higher IOP, more advanced MD, and thinner RNFL (*p* < 0.001). Regional differences were significant for common glaucoma types (*p* = 0.013) and surgical choice (*p* < 0.0001). Late and later-than-optimal referrals ranged between 22% and 38.3%, and 30.0% and 41.7% between the regions, respectively. The proportion of timely referrals in the East European region (20%) was significantly lower than that in the South and West European (approximately 40%) and North European (31.7%) regions (*p* = 0.019).

**Conclusions:**

Marked differences exist in Europe in glaucoma referral timeliness and surgical practice. Late referral remains frequent and associated with advanced structural and functional damage in Europe.

## Key messages

What is known

- Open-angle glaucoma remains a major burden in preserving vision both worldwide and in Europe.
- Successful long-term management of open-angle glaucoma requires effective and sustained intraocular pressure reduction to prevent clinically significant disease progression.
- When achieving sufficiently low intraocular pressure requires incisional glaucoma surgery, an early surgery offers better preservation of visual functions than a late surgery.
- In the past decade referral of open-angle glaucoma patients for glaucoma surgery was predominantly late in Europe, which resulted in development of clinically significant progression during the long pre-surgical glaucoma management.

What is new

- In the current European multicentre investigation, which was conducted in 2025, 300 consecutive open-angle glaucoma referrals from 15 countries, with completed incisional surgical interventions were analysed.
- Despite the increasing surgical options, improved outcomes and reduced postoperative inconvenience with the less invasive techniques the majority of the referrals were still either late (29.7%) or later than optimal (35.7%), and timely referrals with no clinically significant progression during the non-surgical care represented only 34.7%.
- The most common surgeries were trabeculectomy (38.3%), minimally invasive bleb forming surgeries (28.3) and glaucoma drainage device implantations (11.3%).
- Clinically significant differences were found between the East, North, South and West European regions in the timeliness of the referrals, intraocular pressure parameters, visual field deterioration and structural damage, which indicate considerable differences in glaucoma care in Europe in 2025.

## Introduction

Glaucoma remains a major burden of preserving vision in the ageing societies both worldwide and in Europe [1, 2]. In recent years innovations in topical glaucoma medication and increasing use of selective laser trabeculoplasty in open-angle glaucoma has increased the probability of preserving vision and maintaining high vision related quality of life (VRQoL) [3]. Glaucoma surgery, however, remains important intervention to preserve vision in many cases, when glaucomatous progression develops under medical and/or laser treatment [3]. Therefore, it remains essential to refer glaucoma patients for surgery in time, and to avoid unnecessary time delay which may cause additional, functionally significant visual field deterioration and decrease of VRQoL. The appropriateness of referral for glaucoma surgery in Europe was investigated by some of the current authors in 2017 [4]. In that investigation significant differences were found between different geographic regions in Europe. Since 2017 a considerable improvement in the technical and financial conditions has taken place in a wide area in Europe, and the use of various minimally invasive glaucoma surgery (MIGS) and minimally invasive bleb forming glaucoma surgery (MIBS) modalities has gained increasing popularity while trabeculectomy with or without topical or local mitomycin C application remains the gold-standard filtration surgery in open-angle glaucoma [3, 5–7]. In general, most MIGS and MIBS offer clinically significant intraocular pressure (IOP) reduction which is maintained for years, can easily be combined with modern cataract surgery, take less surgical time than trabeculectomy, and interfere less with VRQoL in the early postoperative period [8–12]. Therefore, one may speculate that the current European practice of referral for glaucoma surgery differs from that found by us in the previous decade, while the selected surgical modalities may reflect the current European attitudes in terms of MIGS, MIBS and trabeculectomy preferences.

In order to investigate the characteristics, appropriateness and potential regional differences of the current European practice of referral for glaucoma surgery in open angle glaucoma, and the types of surgery performed, a questionnaire-based investigation was conducted. Each of the participating glaucoma expert groups (one site per country) completed a detailed online questionnaire for 20 consecutive surgical referrals, after surgery in 2025.

## Methods

The research protocol was approved by the Ethical Committee of the Hospital Clinico San Carlos, Madrid, Spain (HCS-DERIV - C.I. 25/198-E). Since this was a retrospective data collection study no consent from patients was obtained. Patients’ personal information was anonymized by the investigators before the data were entered in the questionnaires, after surgery. European glaucoma specialists with high volume glaucoma surgeries (one site per country) were invited to participate in the project, which was conducted between May and September 2025. The EURACCUR study reflects referral patterns observed in tertiary and specialist glaucoma centres and cannot characterize referral behaviour at the total national levels. Referrals for glaucoma surgery to secondary or non-academic care settings were not investigated. Consequently, our findings should be interpreted as referrals pathways representative of tertiary glaucoma specialist clinics rather than comprehensive national practices. Completed online questionnaires for 20 consecutive open-angle glaucoma referrals arrived from fifteen countries (Belgium, Croatia, Denmark, France, Germany, Greece, Italy, Lithuania, Malta, Portugal, Serbia, Slovenia, Spain, Sweden, and The Netherlands). The online questionnaire was supplied with detailed instructions for completion, and comprised seven itemized question groups on patient demographics, type of open-angle glaucoma (primary open-angle glaucoma, POAG; normal tension glaucoma, NTG; pseudoexfoliative glaucoma, PSG; pigmentary glaucoma, juvenile open-angle glaucoma, JOAG, uveitic glaucoma, corticosteroid induced glaucoma, other type), duration of non-surgical glaucoma treatment before the referral, type of the referring practice (university/hospital unit or private practice), IOP before referral for surgery (maximal untreated, typical under treatment and preoperative values, respectively), number of medications for the study eye, visual field and optical coherence tomography (OCT) testing before the referral (yes/no, mean defect/deviation, average retinal nerve fibre layer thickness, RNFLT), stage of glaucomatous deterioration according to the modified Hodapp classification and the current EGS guidelines [3, 13], presence of split fixation, the type of glaucoma surgery (standalone or combined with cataract surgery; MIGS options, MIBS options, trabeculectomy, EX-PRESS^®^ Glaucoma Filtration Device, glaucoma drainage device implantation, nonpenetrating deep sclerectomy (NPDS), ab-externo canaloplasty, cyclodestructive procedures or other interventions), and the investigator’s evaluation of the timeliness of the referral based on preset criteria (timely, later than optimal, late). The questions are presented in Table 1. The number of glaucoma medications for the study eye was expressed in points, summarized from topical medication (1 point for monotherapy, 2 points for fixed dose combinations), oral acetazolamide medication (1 point) and selective laser trabeculoplasty (1 point). Split fixation was defined with no measurable light sensitivity in at least one of the 4 central test point locations for Humphrey perimetry, and at least one of the 5 central locations in Octopus perimetry. A glaucoma referral was considered as “timely” when the progression of glaucoma under treatment was not severe before the referral, or when an immediate referral was made when the diagnosis was made. The referral was considered as “later than optimal” when a clinically significant visual field progression developed during the prior non-surgical care, and “late” when only minimal visual function remained due to progression under non-surgical glaucoma care. The questionnaire captured the overall duration of glaucoma since diagnosis (expressed in months; “duration of glaucoma”). However, it did not include any separate variable specifically addressing the duration of treatment by the first (and any subsequent) managing physician prior to referral, nor the disease stage at first presentation, as data collection focused on the clinical status at the time of referral for surgery. Greece, Italy, Portugal, Spain and Malta were grouped into the “South European” region, Croatia, Serbia and Slovenia into the “East European” region, Belgium, France, Germany, and the Netherlands in the “West-European” region, and Denmark, Lithuania, and Sweden into the “North-European” region.

**Table 1:** Questionnaire items used in the EURACCUR study

Section	Question	Response type/Options
Investigator Data	Country	Open field
	Type of Practice	Public/Private
	Surgeries unavailable in investigator’s practice	Trabecular MIGS/MIBS (Preserflo, Xen)/Express implant/I can use all of them
Patient Data	Gender	Male/Female
	Age at the moment of surgery	Numeric
	Study eye (first operated if both referred)	Right eye/Left eye
	Ethnic origin (self-reported)	Caucasian/African American/Asian/Latino/Other
Glaucoma Diagnosis & History	Type of Glaucoma	POAG/NTG/PSG/Pigmentary/Juvenile/Steroid-induced OAG/Uveitic OAG/Highly myopic glaucoma/Other OAG
	Duration of glaucoma (months)	Numeric
	Type of the referring practice	Hospital–University department/Private practice
Intraocular Pressure	Highest IOP without treatment	Numeric
	Typical treated IOP before surgery	Numeric
	Last IOP prior to surgery	Numeric
Visual Function & Structure	Last visual field performed	Humphrey/Octopus/Other
	MD in last VF before surgery	Numeric
	Split fixation	Yes/No
	Mean RNFL thickness at last OCT before surgery	Numeric
Medical Treatment	Number of medications for the study eye*	Numeric (with detailed instructions)
Glaucoma Severity	Glaucoma severity stage (Modified Hodapp criteria)	Mild/Moderate/Severe
Surgical Data	Type of surgery performed	Trabecular MIGS/Suprachoroidal MIGS/Bleb-forming MIGS (Xen, Preserflo)/Trabeculectomy ± MMC/Express implant/Deep sclerectomy/Ab-externo canaloplasty/glaucoma drainage device (Ahmed, Baerveldt, Paul)/Cyclodestructive surgery/Other
	Combined surgery with phacoemulsification?	Yes/No
Referral Timing	Referral for glaucoma surgery	Timely/Later than optimal/Late

IOP, intraocular pressure; MD, mean deviation; MIBS, minimally invasive bleb forming glaucoma surgery; MIGS, minimally invasive glaucoma surgery; MMC, mitomycin C; OAG, open-angle glaucoma; OCT; optical coherence tomography; POAG, primary open-angle glaucoma; NTG, normal tension glaucoma; PSG, pseudoexfoliative glaucoma; RNFLT, retinal nerve fibre layer thickness;

* The number of glaucoma medication for the study eye is expressed in points, summarized from topical medication (1 point for monotherapy, 2 points for fixed dose combinations), oral acetazolamide (1 point) and selective laser trabeculopasty (1 point)

### Statistics

Analyses were performed with SPSS version 29.0.2 (IBM Corp., Armonk, NY, USA). Continuous variables were initially assessed for normality using the Shapiro–Wilk test. As all continuous variables significantly deviated from a normal distribution (*p* < 0.05 for all), non-parametric statistical methods were applied. Continuous variables are presented as median and quartiles when appropriate, whereas categorical variables are expressed as absolute frequencies and percentages.

Comparisons of continuous variables across more than two independent groups (e.g., geographic regions, glaucoma subtypes, or surgical techniques) were made using the Kruskal–Wallis test. When global significance was detected, post hoc pairwise comparisons were performed using Dunn’s test with Bonferroni correction. Distributions of categorical variables, such as glaucoma type, surgical technique, or timeliness of the referral, were evaluated using Pearson’s chi-square test; when more than 20% of expected cell counts were below five Yates’s continuity correction or Fisher’s exact test was applied as appropriate.

When visual field testing was made with an Octopus perimeter, the mean defect value was converted to Humphrey mean deviation (MD), and the calculations were made with the Humphrey MD values. Key clinical parameters, including IOP at different time points, average RNFL thickness, visual field MD, and duration of glaucoma, were specifically analysed according to type of surgical procedure, and geographic region. Additional analyses were performed to evaluate the relationship between referral timeliness and markers of structural and functional severity, using both non-parametric tests and chi-square statistics where applicable. Effect sizes are reported using H, U, or χ² values together with the corresponding p-values. Where appropriate, effect sizes were calculated to complement p-values and facilitate interpretation of clinical relevance. All statistical tests were two-tailed. A p-value < 0.05 was considered statistically significant.

## Results

A total of 300 eyes of 300 consecutive patients referred for glaucoma surgery were analysed. Referrals from hospital- or university-based ophthalmology departments and private practices represented 64.3% and 35.7%, respectively. No difference in the distribution of referrals was found between the geographic regions (χ² = 2.05; *p* = 0.56). The median (quartiles) age of the total cohort at the time of surgery was 72 years (64–78), ranging from 23 to 94 years (Table 2). The study population was predominantly Caucasian (95.0%), reflecting the demographic profile of the participating centres, with a minority representation of African American, Latino, Asian, and other ethnic backgrounds. Regarding the type of open-angle glaucoma, the most frequent diagnosis was POAG, accounting for 52.0% of cases, followed by PSG (23.0%), other secondary open-angle glaucomas (9.3%), NTG (5.0%), pigmentary glaucoma (3.0%), uveitic glaucoma (3.0%), and juvenile glaucoma (2.3%).

**Table 2:** Clinical characteristics of the total study sample

Characteristics at the time of referral: total population, median (quartiles) and range	
Sex (%)	Male: 56% — Female: 44%
Age at surgery (years)	Median 72 (64–78), range 23–94
Glaucoma type (%)	POAG 52%; PSG 23%; Secondary open angle, not PSG 17.7%; NTG: 5%; Juvenile 2.3%
Duration of glaucoma (months)	Median 75 (28–144), range 1–420
Highest untreated IOP (mmHg)	Median 29 (24–37), range 12–57
Typical treated IOP (mmHg)	Median 20 (18–26), range 4–65
Preoperative IOP (mmHg)	Median 22 (18–28), range 10–65
Number of glaucoma medications, for the study eye*	Median 4 (3–4), range 0–7
Mean deviation (MD, dB)	Median − 14.47 (− 21.60 to − 7.85), range − 33.42 to − 2.93
Split fixation	30%
RNFL thickness (µm)	Median 60 (50–75), range 28–111

IOP, intraocular pressure; MD, mean deviation; RNFL thickness, retinal nerve fibre layer thickness measured with optical coherence tomography

POAG: primary open angle glaucoma, PSG: pseudoexfoliative glaucoma, NTG: normal tension glaucoma

* The number of glaucoma medication for the study eye is expressed in points, summarized from topical medication (1 point for monotherapy, 2 points for fixed dose combinations), oral acetazolamide (1 point) and selective laser trabeculopasty (1 point)

The time elapsed since diagnosis showed considerable variability, with a median of 75 months (quartiles 28–144 moths) and a range of 1 to 420 months. The maximum untreated IOP was 29 mmHg (24–37; range: 12–57 mmHg); the most frequently recorded treated IOP was 20 mmHg (18–26; range: 4–65 mmHg), and the preoperative IOP was 22 mmHg (18–28; range: 10–65 mmHg). Patients were receiving a median of 4 (3–4; range: 0–7) hypotensive medications before surgery.

Most patients had structural and functional assessments available at the time of inclusion: 92.7% had a recent visual field test and 88.3% had an OCT RNFL measurement documented. The preoperative MD was − 14.47 dB (− 21.60 to − 7.85; range: −2.93 to − 33.42 dB), and the average RNFL thickness was 60 μm (50–75; range: 28–111 μm), both of which reflect advanced functional and structural damage.

Regarding surgical intervention, the most frequently performed procedure in the total population was trabeculectomy with or without mitomycin C, used in 38.3% of patients. MIBS (Xen^®^ Gel Stent: Allergan, an AbbVie Company; Irvine, CA, USA; and Preserflo^®^ MicroShunt: Santen Pharmaceutical; Osaka, Japan) accounted for 28.3%, while drainage devices (Ahmed^®^ Glaucoma Valve, New World Medical; Rancho Cucamonga, CA, USA; Baerveldt^®^ Glaucoma Implant, Johnson & Johnson Surgical Vision; Santa Ana, CA, USA; and the Paul^®^ Glaucoma Implant, Advanced Ophthalmic Innovations; Singapore) were used in altogether 11.3% of cases. Trabecular MIGS represented 8.7%, followed by EX-PRESS^®^ Glaucoma Filtration Device (Alcon Laboratories, Fort Worth, TX, USA) implantation (5.0%), nonpenetrating deep sclerectomy (4.3%), and cyclodestructive procedures (3.3%). No other types of glaucoma surgical interventions occurred. Only 18.7% of surgeries were combined with phacoemulsification The type of surgery was strongly related to disease severity categories, such that bleb forming filtering and drainage procedures were preferentially used in patients with poorer prior IOP control and more advanced damage, whereas trabecular MIGS were predominantly performed in eyes with less elevated IOP (χ² = 93.3; *p* < 0.001, Cramér’s V = 0.56).

Assessment of referral timeliness in the total population showed that 35.7% of patients were referred later than optimal, and 29.7% were referred late, with severely limited visual field deterioration. Only 34.7% were referred in a timely manner, before clinically significant deterioration developed. Late referrals exhibited substantially worse functional loss (Kruskal–Wallis H = 124.9, *p* < 0.001, ε² = 0.42) and thinner RNFL (H = 29.8, *p* < 0.001). The proportion of eyes showing split fixation increased markedly with referral delay, from approximately 10% among timely referrals to 28% among those referred later than optimal and 42% among those referred late.

When stratifying the cohort by European geographical regions (Table 3), 100 patients were from South Europe, 80 from West Europe, 60 from East Europe, and 60 from North Europe. Age differed significantly across regions (Kruskal–Wallis H = 18.77; *p* = 0.0003), being lower in East Europe and higher in North Europe. The duration of glaucoma did not differ significantly across regions (Kruskal–Wallis *p* = 0.106). However, the IOP parameters showed significant regional variability.

**Table 3:** Clinical and demographic characteristics in the European regions

Characteristics (%, or median and range)	South Europe (1)	East Europe (2)	West Europe (3)	North Europe (4)	*P*-value	1 vs. 2	1 vs. 3	1 vs. 4	2 vs. 3	2 vs. 4	3 vs. 4
Sex (%, male)	56%	55%	58%	53%	0.842*						
Age (years)	72 (64–78)	68 (59–75)	70 (63–79)	74 (66–80)	0.0003†	0.018	0.412	0.009	0.041	0.001	0.067
Most frequent glaucoma type (%)	PSG (38%)	POAG (52%)	POAG (48%)	POAG (50%)	0.013*	0.090	0.012	0.260	0.006	0.036	0.110
Duration of glaucoma (months)	78 (30–150)	70 (24–120)	82 (30–140)	75 (29–150)	0.106†						
Highest untreated IOP (mmHg)	30 (26–38)	28 (24–36.2)	26.5 (22–32)	33.5 (24.5–40.2)	0.024†	0.142	0.021	0.118	0.048	0.071	0.004
Typical treated IOP (mmHg)	20 (18–25)	22.5 (18.7–26)	17 (15–21)	24 (19.2–31.5)	< 0.001†	0.030	< 0.001	0.012	< 0.001	0.046	< 0.001
Preoperative IOP (mmHg)	24 (21–30.2)	22 (18–30)	18 (15.7–21.2)	22.5 (17–27.2)	< 0.001†	0.064	< 0.001	0.083	< 0.001	0.112	< 0.001
RNFL thickness (µm)	64.4 (50–75)	69.1 (50–85)	58.2 (48–66)	60.3 (47–72)	< 0.001†	0.021	0.008	0.334	< 0.001	0.004	0.041
MD (dB)	–13.3 (–21.6 to − 7.8)	–16.7 (–22.5 to − 10.1)	–14.8 (–22.0 to − 8.3)	–12.2 (–18.6 to − 6.9)	0.055†						
Split Fixation (%)	27%	39%	31%	29%	0.190*						

IOP, intraocular pressure; M, male sex; MD, mean deviation; RNFL thickness, retinal nerve fibre layer thickness measured with optical coherence tomography

* χ² test and pairwise χ² test with Bonferroni-correction

† Kruskal–Wall test with Dunn’s post-hoc test and Bonferroni correction

Maximum untreated IOP differed across regions (Kruskal–Wallis *p* = 0.024) with median (quartiles) values of 30 mmHg (26–38) in South Europe, 28 mmHg (24.0–36.2) in East Europe, 26.5 mmHg (22–32) in West Europe, and 33.5 mmHg (24.5–40.2) in North Europe (Table 4).

Typical treated IOP also differed significantly across regions (Kruskal–Wallis *p* < 0.001), being lowest in West Europe (17 mmHg; 5–21) and highest in North Europe (24 mmHg; 19.2–31.5), with intermediate values in South Europe (20 mmHg; 18–25) and East Europe (22.5 mmHg; 18.7–26.0). The preoperative IOP also demonstrated significant inter-regional differences (Kruskal–Wallis *p* < 0.001), with the highest values observed in South Europe (24 mmHg; 21.0–30.2) compared with East Europe (22 mmHg; 18–30), North Europe (22.5 mmHg; 17.0–27.2), and the lowest in West Europe (18 mmHg; 15.7–21.2).

The distribution of glaucoma types also differed between the regions (χ² = 41.8; *p* = 0.013). The South-European region was characterized by a higher prevalence of PSG, the East-European and the West-European by a predominance of POAG, and the North-European by a mix of POAG and PSG.

Significant regional differences were also observed in the choices of surgery (χ² = 170.3; *p* < 0.001). In the East European region, traditional trabeculectomy was predominantly used (63.3%), together with a substantial proportion of EX-PRESS Device procedures (25.0%). In the South-European region, a mixed surgical profile was observed, combining filtering procedures (38.0%), MIBS options (34.0%) and drainage devices (20.0%). In the West European region, trabecular MIGS and less invasive options were preferred, with 22.5% of the eyes undergoing trabecular MIGS and 30.0% MIBS options, accompanied by a smaller proportion of trabeculectomy (16.3%) and glaucoma drainage device surgery (15.0%). In the North European region, a more balanced pattern was observed, with a predominance of trabeculectomy (43.3%) and MIBS options (23.3%), while other techniques were rarely used.

Referral timeliness was first analysed using the three predefined categories (timely, later than optimal, and late). When considering the full distribution, a non-significant trend toward regional differences was observed (χ², *p* = 0.055), suggesting heterogeneous referral patterns in Europe without reaching formal statistical significance after correction for multiple comparisons. To further explore clinically relevant differences, referral timing was subsequently dichotomized as timely versus non-timely (later than optimal and late in combination). This analysis revealed significant regional differences (χ², *p* = 0.019), with East Europe showing the lowest proportion of timely referrals (20%), compared with South and West Europe (approximately 40%), and North Europe (31.7%).

**Table 4:** Comparison of timeliness of the referrals between the European regions (%)

	South Europe	East Europe	West Europe	North Europe	*P*-value
Timely	41.0 %	20.0 %	40.0 %	31.7 %	0.055
Later than optimal	37.0 %	41.7 %	33.8 %	30.0 %	
Late	22.0 %	38.3 %	26.2 %	38.3 %	

χ^2^ test

When analysing functional and structural status across referral categories, significant differences were observed. Late-referred patients showed markedly worse visual field MD (Kruskal–Wallis H = 124.9, *p* < 0.001) and significantly thinner RNFL (H = 29.78, *p* < 0.001) compared with those referred on time or later than optimal. In contrast, intraocular pressure parameters, including maximum untreated IOP, typical treated IOP, and preoperative IOP, did not differ significantly across referral categories (*p* > 0.100 for all).

## Discussion

### Comparison with prior evidence and epidemiological context

The EURACCUR Study aims to provide a contemporary European overview of open-angle glaucoma referral patterns and surgical glaucoma practice in 2025. Therefore, centres from several, geographically different European countries were invited to participate in the project. Fiveteen of these countries completed the questionnaire for 300 consecutive open-angle glaucoma patients in a distribution that made it possible to establish four European “regions”. In order to create sufficiently powered datasets for meaningful within-Europe comparisons, geographically proximate countries with shared historical and broad socioeconomic characteristics were grouped into European geographic regions. This grouping strategy was adopted to enhance statistical robustness while reflecting common structural features of healthcare delivery across neighbouring countries. However, these regional groupings do not imply homogeneity in healthcare organization, reimbursement policies, access to diagnostic technologies, or availability of surgical devices, which may vary substantially between countries even within the same geographic region.

In our cohort advanced functional and structural damage at the time of referral was overrepresented, despite maximal medical therapy. This finding is consistent with the increasing epidemiological burden of glaucoma projection models for Europe, which estimate more than 12 million affected individuals in 2024 and anticipate a further rise by 2050 due to demographic ageing [1, 2]. Importantly, more than half of glaucoma cases in Europe remain undiagnosed in the general population [2], suggesting that many patients reach ophthalmology care, and ultimately surgical referral, but at relatively advanced stages.

The predominance of POAG and PSG in our cohort aligns with the updated European Eye Epidemiology Consortium findings [2], as well as earlier systematic reviews showing that POAG remains the dominant subtype in the Western and Northern regions [14]. The advanced structural damage and significant visual field deterioration at the time of referral for glaucoma surgery found in the EURACCUR study reflect this epidemiologic trend, particularly in regions where PSG is more aggressive and faster-progressing, even if potential differences in the quality of glaucoma care during the non-surgical treatment period could also play a role in the outcome. These potential differences, however, were not investigated in the current study.

Earlier European investigations highlighted persistent deficiencies in detecting structural progression and integrating OCT-based analysis into routine glaucoma surveillance [4, 15]. Despite substantial advances in OCT technology, introducing widefield imaging and artificial intelligence (AI)-assisted progression tools in glaucoma care over the last decade, our present results suggest that late surgical referral continues to occur at a high rate, indicating that these technologies are yet to be widely implemented in routine clinical practice. A large proportion of clinically significant visual field deterioration developed before referral for surgical intervention in the EURACCUR study. This raises important concerns regarding the translation of technological advances and guidelines into real-life clinical practice [3, 16].

The relevance of appropriate timing of referral has been well documented, as progression of glaucomatous field loss increases the risk of falls, reduced reading ability and leads to impairment of VRQoL [17–20]. In this context, the high proportion of late and later-than-optimal referrals in the EURACCUR study underscores a persistent clinical gap with substantial functional consequences.

### Regional differences in clinical presentation and its relevance to clinical practice

Preoperative IOP differed significantly across four European regions, with the highest median values in South Europe (24 mmHg) and the lowest in West Europe (18 mmHg), followed by East Europe (22 mmHg) and North Europe (22.5 mmHg).

Structural and functional damage also differed across regions. The median RNFL thickness was highest in East Europe (69.1 μm) and lowest in West Europe (58.2 μm) showing that the structural damage was typically advanced in all regions when the referral for glaucoma surgery was made. The apparent discordance between structural and functional measures, particularly the relatively higher mean RNFL thickness observed in East Europe despite the worse visual field MD, is explained by differential availability of OCT data. In routine clinical practice, OCT imaging is often omitted in eyes with advanced glaucoma, since RNFL thickness measurements are subject to floor effect, and provide limited additional clinical information. In contrast, visual field testing remains a useful tool even in advanced glaucoma. Consequently, RNFL thickness data are more likely to be available for eyes with mild and moderate structural damage, leading to a virtual overestimation of average RNFL thickness in geographic regions where the regional visual field deterioration is advanced.

The distribution of functional loss followed a pattern, in which more pronounced MD deterioration was found in East and West Europe (median − 16.7 dB and − 14.8 dB, respectively) compared with South Europe (− 13.3 dB) and North Europe (− 12.2 dB). However, these median values all show that the typical functional deterioration was advanced in each region when glaucoma surgery was considered. The proportion of timely referrals varied markedly, being highest in South and West Europe (41% and 40%, respectively), somewhat lower in North Europe (31.7%), and considerably lower in East Europe (20%). These figures, however, show that even in the best performing regions the majority of the cases were not referred for surgery in time.

These geographic disparities found by us mirror the findings of a recent European task-force analysis, which highlighted substantial heterogeneity in diagnostic infrastructure, access to OCT and visual field devices, and availability of minimally invasive glaucoma surgical technologies [21]. The present study reinforces that structural differences between healthcare systems may translate into clinically relevant discrepancies in disease severity at the time of referral for glaucoma surgery.

### Patterns of surgical choice and their determinants

Selection between choices of surgeries for open-angle glaucoma in the EURACCUR study varied widely among regions. Trabeculectomy remains the most frequently performed procedure overall, reflecting its well-documented long-term IOP-lowering efficacy [6, 7]. However, the expansion of MIBS options, and the recent evidence supporting their safety and effectiveness [22–26], explain the increasing popularity of these options in Europe. In our investigation the highest rate of using trabecular MIGS was found in the West-European region. This suggests a broader device availability and affordability, which may increase the glaucoma surgeons’ familiarity with the Schlemm’s canal-based approaches. The East-European region relied predominantly on trabeculectomy, reflecting more traditional surgical paradigms and possibly limited access to and affordability of newer and more expensive technologies. In the South-European region, where the disease severity was particularly high in our investigation, a similar use of trabeculectomy, MIBS and drainage devices were preferred. It is important to note that economic factors could also play a role in the choice of surgery. Significant differences in device availability/affordability, national reimbursement policies, private insurance coverages and procedural costs exist in the various European countries. However, as detailed information on surgical costs and reimbursement structures was not collected in the present study, the impact of economic factors on the procedure selection cannot be quantified. Therefore, the results need to be interpreted in the context of heterogeneous healthcare financing systems across Europe.

Clinical trial data demonstrate that MIBS often offer substantial IOP reduction with faster postoperative visual recovery compared with trabeculectomy [8–11, 26]. However, these benefits are primarily relevant in patients who are referred for surgery early, long before the late stage structural and functional damage develops. Unfortunately, considering the total EURACCUR population the majority of patients were referred in a glaucoma stage which was too late to benefit from the less invasive options, reinforcing the deleterious impact of delayed referral.

### Referral timing and consequences of delay

One of the most striking findings of the EURACCUR study is the high proportion of delayed referrals, in all European regions. Late-referred patients showed substantially more visual field deterioration and lower RNFL thickness compared to those with timely referrals. In the current investigation the success of glaucoma surgery was not investigated. However, our results also suggest that prolonged topical treatment, particularly when involving preserved eye drops, may compromise conjunctival health and reduce surgical success rates [27].

Late referral also limits the range of viable surgical options. Although MIGS were fond effective in mild-to-moderate glaucoma [5, 8], eyes with advanced disease often require filtration surgery or glaucoma drainage device implantation. The more aggressive procedures carry a higher risk of early postoperative hypotony, slower visual recovery and a greater likelihood of bleb-related complications [6, 7, 22, 23].

The persistence of late referral patterns in Europe in 2025 suggests systematic barriers, including fragmented care pathways, limited access to and use of imaging technologies, financial limitations to widely access the MIGS and MIBS options, and incomplete adoption of structured progression-monitoring protocols. The potential underuse of selective laser trabeculoplasty as a first-line therapy, as reported in an earlier European study, may also contribute to delayed surgical decision-making [4]. In addition, neither physician-related variables of the referring ophthalmologists (e.g. experience or subspecialty training), nor patient-related socioeconomic factors were evaluated. Patient-related factors could also contribute to delayed referrals. In particular, poor adherence to the prescribed medication and/or scheduled follow-up visits could result in a delayed detection of progression, and as a consequence, a less than optimal timing of referral for surgery. Since patient compliance at the referring setting and during any earlier glaucoma care periods was not investigated by us, its impact could not be analysed in the current study.

The observed distribution of surgical procedures does not exclusively reflect clinical disease severity. Although our questionnaire captured the procedures and recorded whether certain surgical options were unavailable in the investigators’ practices, it did not assess surgeons’ preferences, intentional decision-making pathways, or detailed device availability constraints at the individual patient level. Consequently, variation in surgical choices may also reflect health care system related factors such as access, reimbursement and institution availability. To consider these divers factors additional variables are required, which were not collected in our investigation.

### Strengths and limitations

The strengths of the EURACCUR study include its real-world, multicentre European design, standardization of data collection, and the ability to assess both clinical severity and surgical patterns across diverse healthcare systems in 2025. The questionnaire used in the current investigation is similar to that applied in an earlier European study (the Ref-GS study) which investigated referrals for glaucoma surgery in 2017 [4]. Even if the results of the two investigations conducted 8-years apart are not directly comparable, the current EURACCUR study did not find a major improvement in the timeliness of the referrals for glaucoma surgery in Europe. This outcome is particularly worrying since update editions of the European Glaucoma Society Guidelines, which all support timely glaucoma surgery, have been freely available and downloadable in various European languages for decades.

Our study has limitations. We could not address the influence of care quality during the pre-referral period on the outcome. Findings are representative of tertiary specialist referral pathways rather than national referral practice. The number of cases analysed by us made it possible to compare European “regions” and detect significant differences between these regions even after Bonferroni correction. But the 300 referrals we collected may not be fully representative for the real, larger European regions, which include countries from which no data relevant to the current investigation arrived. Our study design does not allow us to assess the relationship between the duration of care by the first (and any subsequent) physician, and the stage of glaucoma at the initial presentation. The EURACCUR Study focused on disease severity at the time of referral for glaucoma surgery. Thus, associations between disease stage, duration of initial management, and later referral timing cannot be analysed using the present dataset. Another limitation relates to the regional grouping strategy. Although countries were grouped to allow comparative analyses, substantial heterogeneity exists within the regions regarding healthcare systems, reimbursement structures, access to diagnostic tools and availability of glaucoma surgical technologies. These within-region differences may not be fully captured by broad geographic grouping while they could influence referral patterns and surgical choices. Therefore, the observed regional differences should be interpreted as descriptive and comparative associations, rather than clear causal relationships.

Further, a larger sample size might have increased the probability of identifying additional significant between-region differences. In the current investigation the main goal was to evaluate referral timeliness for various European areas. To facilitate this, we used an easy-to-apply, uniform disease severity category distribution, which can be refined for similar future investigations focusing on smaller geographic areas, where glaucoma patients are tested with less diverse perimetry and OCT systems.

### Future directions

Future work should prioritize continuous education on measuring and understanding glaucomatous progression, integration of structural and functional progression analyses, particularly trend-based OCT and visual field progression analyses for routine glaucoma management in Europe. The expansion of teleophthalmology models and centralized diagnostic hubs could mitigate regional disparities in diagnostic infrastructure. Improving access to selective laser trabeculoplasty and MIGS/MIBS in regions where these modalities remain underutilized may allow earlier non-surgical and surgical interventions and reduce reliance on conventional filtration surgery. Artificial intelligence-driven progression algorithms hold promise for prompting earlier detection of deterioration and standardizing referral thresholds. Harmonized European referral pathways and reimbursement models could also further reduce the geographic variability.

## Conclusions

Despite advances in diagnostic technologies, medical education tools and the expansion of minimally invasive surgical options, late referral for glaucoma surgery remains a major challenge in Europe. The EURACCUR Study highlights substantial regional heterogeneity in disease phenotype, clinical severity at the time of referral for surgery, and considerable differences in surgical practice. Addressing systemic barriers to early detection, standardizing progression assessment and harmonizing referral criteria in Europe are essential to reduce the preventable decline of VRQoL and optimize glaucoma care.

## Data Availability

No datasets were generated or analysed during the current study.

## Declarations

The research protocol was approved by the Ethical Committee of the Hospital Clinico San Carlos, Madrid, Spain (HCS-DERIV - C.I. 25/198-E). Since this was a retrospective data collection study no consent from patients was obtained. Patients’ personal information was anonymized by the investigators before data were entered in the questionnaires, after surgery.
